# S-layers: The Proteinaceous Multifunctional Armors of Gram-Positive Pathogens

**DOI:** 10.3389/fmicb.2021.663468

**Published:** 2021-04-06

**Authors:** Janani Ravi, Antonella Fioravanti

**Affiliations:** ^1^Pathobiology and Diagnostic Investigation, Michigan State University, East Lansing, MI, United States; ^2^Structural and Molecular Microbiology, VIB-VUB Center for Structural Biology, Brussels, Belgium; ^3^Structural Biology Brussels, Vrije Universiteit Brussel, Brussels, Belgium

**Keywords:** gram-positive bacteria, Firmicutes, cell envelope, S-layer, cell surface proteins, pathogenicity, sequence-structure features, molecular evolution

## Abstract

S-layers are self-assembled crystalline 2D lattices enclosing the cell envelopes of several bacteria and archaea. Despite their abundance, the landscape of S-layer structure and function remains a land of wonder. By virtue of their location, bacterial S-layers have been hypothesized to add structural stability to the cell envelope. In addition, S-layers are implicated in mediating cell-environment and cell-host interactions playing a key role in adhesion, cell growth, and division. Significant strides in the understanding of these bacterial cell envelope components were made possible by recent studies that have provided structural and functional insights on the critical S-layer and S-layer-associated proteins (SLPs and SLAPs), highlighting their roles in pathogenicity and their potential as therapeutic or vaccine targets. In this mini-review, we revisit the sequence-structure-function relationships of S-layers, SLPs, and SLAPs in Gram-positive pathogens, focusing on the best-studied classes, Bacilli (*Bacillus anthracis*) and Clostridia (*Clostridioides difficile*). We delineate the domains and their architectures in archetypal S-layer proteins across Gram-positive genera and reconcile them with experimental findings. Similarly, we highlight a few key “flavors” of SLPs displayed by Gram-positive pathogens to assemble and support the bacterial S-layers. Together, these findings indicate that S-layers are excellent candidates for translational research (developing diagnostics, antibacterial therapeutics, and vaccines) since they display the three crucial characteristics: accessible location at the cell surface, abundance, and unique lineage-specific signatures.

## Introduction

Prokaryotes have evolved sophisticated and multi-layered cell envelopes to protect them while allowing selective cell-environment trafficking of nutrients, metabolites, integration of signals, and release of effectors. Despite the enormous diversity observed among prokaryotes and their environmental niches, the most commonly observed outermost cell envelope component is the surface layer (S-layer; Sleytr and Beveridge, 1999; Sara and Sleytr, 2000; Albers and Meyer, 2011; Fagan and Fairweather, 2014; Rodrigues-Oliveira et al., 2017). S-layers are semipermeable (glyco-) protein monolayers formed by S-layer proteins (SLPs) that once released at the cell surface self-assemble into a paracrystalline 2D lattice with defined symmetry that is anchored at the cell envelope. S-layers demand a high metabolic investment from the organism producing them; they comprise 5–15% of the total cellular protein production, making them amongst the most abundant proteins on Earth (Sara and Sleytr, 2000).

Since their first observation in the 1950s, the biological and biotechnological relevance of S-layers has been of great interest to the scientific community (Sleytr et al., 2014). Nevertheless, half a century later, even though S-layers are found nearly in all major bacterial clades and represent an almost universal feature of the archaeal cell envelope, our knowledge about their structure and function remains patchy. Multiple factors have contributed to this lack of knowledge: (i) the absence of S-layers in classical model organisms such as *Escherichia coli* and *Bacillus subtilis*; (ii) the self-assembling characteristic of SLPs, that has long hampered structural and biochemical studies; and (iii) their low sequence homology, making it challenging to identify S-layer-carrying organisms based on their sequence alone. Function-wise, while the SLPs in archaea are known to maintain cell shape, often as the sole cell-wall components (Albers and Meyer, 2011; Rodrigues-Oliveira et al., 2017), bacterial S-layers are known to carry out multiple functions ranging from adhesive surface to protective and selective barriers (Gerbino et al., 2015).

Several Gram-positive and negative pathogens possess S-layers that play potentially significant roles in their virulence (Carl and Dasch, 1989; Kawai et al., 1998; Mignot et al., 2001; Thompson, 2002; Shimotahira et al., 2013; Rasmussen-Ivey et al., 2016). In this mini-review, we provide a comprehensive overview of the current understanding of S-layer structure, function, and contribution to the pathogenicity of Gram-positive bacteria, focusing on the best-characterized S-layer-carrying human pathogens: *Bacillus anthracis* and *Clostridioides difficile*. We discuss recent breakthroughs on the S-layer structure and function of these two pathogens that emphasize the role of SLPs as promising antimicrobial targets (Kirk et al., 2017; Fioravanti et al., 2019; Oatley et al., 2020; Banerji et al., 2021). Along with highlighting variations seen across Firmicutes and Actinobacteria, this review provides a foundation and context for future studies to fully exploit the potential of SLPs as targets for the development of novel diagnostics, vaccines, and antibacterial therapies.

## Two Armors Are Better Than One? the *B. anthracis* S-layer Case

*Bacillus anthracis* is the etiological agent of anthrax (Kamal et al., 2011; Goel, 2015; Okinaka and Keim, 2016) and a CDC Category A bioterrorist agent [Centers for Disease Control and Prevention (CDC), 2018]. As part of its immune evasion strategy, this sporulating bacterium, displays a complex and dynamic cell envelope composition (Chateau et al., 2020) that includes switchable S-layers (Mignot et al., 2002).

### The Two Switchable S-layers, Sap and EA1

On top of a thick peptidoglycan layer (PG), the bacterium cell surface is enveloped by one of two mutually exclusive S-layers, the Sap or EA1 S-layer, present, respectively, during exponential and stationary growth phase of cells grown in rich medium (Mignot et al., 2002, 2003; Fioravanti et al., 2019). Electron microscopy (EM) observation of single S-layer mutants revealed a clear difference between the 2D arrays: the Sap S-layer forms a continuous array, whereas the EA1 S-layer is organized in patches (Couture-Tosi et al., 2002). The SLPs developmental switch is controlled by growth-phase-specific sigma factors and the two SLPs (Mignot et al., 2002), which contain *C*-terminal domains with DNA-binding activity that independently repress the *eag* promoter (Couture-Tosi et al., 2002; Mignot et al., 2002). To ensure high expression, bacterial SLPs are associated with strong promoters, efficient transcription, and mRNAs with increased stability (∼6–10x in *B. anthracis* SLPs compared to the average bacterial mRNA half-life; Glatron and Rapoport, 1972; Fisher et al., 1988; Emory et al., 1992; Mignot et al., 2002). The S-layer switch also occurs during systemic infection as both proteins are immunogenic during human anthrax infection (Baillie et al., 2003). It is still unclear why *B. anthracis* performs this energetically expensive S-layer remodeling during its life cycle and infection, emphasizing the need to understand environmental and host triggers that induce this switch.

### Sequence-Structure Features

**FIGURE 1:**
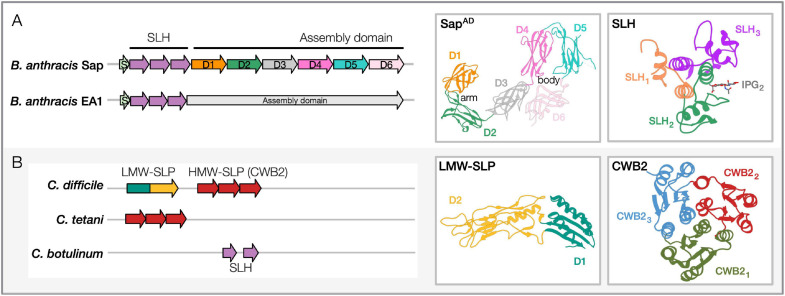
Sequence-structure features of pathogenic Bacilli and Clostridia SLPs. **(A)** *Bacillus anthracis* SLPs, Sap and EA1. Domain architectures: SLH region (containing three SLH domain motifs, Pfam PF00395, ProSiteProfiles PS51272) and the assembly domain are shown for both the representative Bacillus SLPs. PDB structures: Shown for Sap^AD^ and Sap-SLH are shown and labeled [PDB: 6HHU, 6BT4 (Kern et al., 2011; Fioravanti et al., 2019)]. The crystal structure of the SLH domain is in complex with a synthetic SCWP unit (IPG). Accession numbers for proteins shown: Sap, AAT53168.1; EA1, AAP24884.1. Similar domain architectures are observed in *B. cereus*, *B. mycoides*, and *B. thuringiensis* SLPs. **(B)** Clostridia SLPs in *Clostridioides difficile*, *Clostridium tetani*, and *Clostridium botulinum*. Domain architectures: *C. difficile* contains low-molecular-weight (LMW) and high-molecular-weight (HMW) SLP domains (HMW-SLP with three CWB2 domain motifs; LMW-SLP). *C. tetani* contains the cell-wall binding domain (CWB2, Pfam PF04122) as well, while *C. botulinum* carries two SLH domains. PDB structures: Shown for the LMW-SLP and CWB2 of Cwp8 are shown [PDB: 3CVZ, 5J6Q; (Fagan et al., 2009; Usenik et al., 2017)]. Accession numbers for proteins shown: *C. difficile*, WP_078051019.1; *C. tetani*, WP_035111087.1; *C. botulinum*, WP_039307708.1. Domain architectures are marked from cited literature and InterProScan predictions (Jones et al., 2014). Representative PDB structures for Bacillus and Clostridia SLP domains have been redrawn using Phyre2 (Kelley et al., 2015).

**FIGURE 2:**
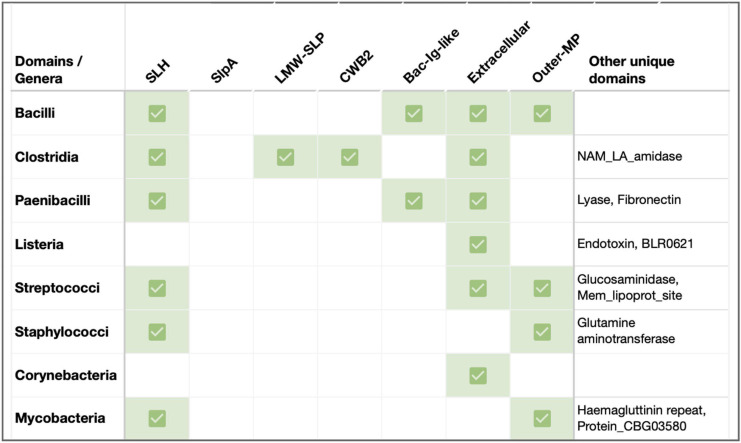
Sequence features of SLPs in key Gram-positive pathogenic genera. Previously documented or predicted [using InterProScan (Jones et al., 2014)] sequence-structure features in representative SLPs from select pathogens in Firmicutes and Actinobacteria. Species considered: *B. anthracis*, *B. cereus*, *B. mycoides*, *B. thuringiensis*, *Clostrioides difficile*, *Clostridium tetani*, *Clostridium botulinum*, *P. alvei*, *Listeria monocytogenes*, *L. seeligeri*, *L. booriae*, *L. fleischmannii*, *Streptococcus pneumoniae*, *S. dysgalactiae*, *S. pyogenes*, *Staphylococcus cohnii*, *S. haemolyticus*, *Corynebacterium glutamicum*, *C. aurimucosum*, *C. minutissimum*, *M. intracellulare*, and *M. kansasii*. Abbreviations: *SLH*, S-layer homology domain (Pfam PF00395, ProSiteProfiles PS51272); *LMW-SLP*, Low molecular weight S layer protein *N*-terminal (Pfam PF12211); *CWB2*, Cell wall binding domain 2 (High molecular weight; Pfam PF04122); Extracellular, Region of a membrane-bound protein predicted to be outside the membrane in the extracellular region (Phobius prediction); Outer-MP, Outer membrane protein alpha-related (PANTHER PTHR43308); *Bac-Ig-like*, Bacterial Ig-like domain (Pfam clan CL0159: PF02368, PF13205); Lyase, Hyaluronate lyase/Polysaccharide lyase family 8 (Pfam PF02278, PF02884, PF08124, PANTHER PTHR38481); Fibronectin, Fibronectin type-III domain (Pfam PS50853); *NAM_LA_amidase*, N-acetylmuramoyl-L-alanine amidase-related domain (PANTHER PTHR30032); *Glucosaminidase*, Mannosyl-glycoprotein endo-beta-N-acetylglucosaminidase, Transglutaminase-like superfamily (Pfam PF01832, PF01841); *Mem_lipoprot_site*, Prokaryotic membrane lipoprotein lipid attachment (ProSiteProfiles PS51257); *Glutamine aminotransferase*, Glutamine amidotransferase type 2; Glucosamine-fructose-6-phosphate aminotransferase, isomerizing (Pfam PF13522, ProSiteProfiles: PS51278, PANTHER PTHR10937); and Endotoxin, Delta endotoxin (Pfam PF18449).

The two SLPs contain similar domain architectures that include: an *N*-terminal signal peptide for secretion, an S-layer homology (SLH) domain for cell anchoring, and a *C*-terminal assembly domain (AD) that self-assembles into the S-layer (Figure 1A; Mesnage et al., 1999; Candela et al., 2005; Wang et al., 2015). Given their abundance, both SLPs are secreted by an accessory and dedicated secretion system (Nguyen-Mau et al., 2012). Once released at the cell surface, they spontaneously fold and anchor at the cell wall through non-covalent interactions between the SLH domain and the pyruvylated secondary cell wall polysaccharides (SCWP) bound to the PG (Mesnage et al., 2000; Kern et al., 2010; Missiakas and Schneewind, 2017). SCWP is essential for cell growth and division and plays a critical role in bacterial pathogenicity (Oh et al., 2016; Chateau et al., 2018). The SLH-SCWP interaction is considered an ancestral mechanism for SLP anchoring to the cell envelope (Cava et al., 2004), and in Gram-positive bacteria, it is recurrent in cell-wall-anchored proteins (Figure 2). *B. anthracis* additionally encodes 22 S-layer-associated proteins (SLAPs, called BSLs in Bacilli) that harbor SLH domains (Kern and Schneewind, 2008). Unlike SLPs, BSLs are minor components of the envelope that do not form paracrystalline arrays but exploit several enzymatic functions participating in different cellular processes [e.g., peptidoglycan metabolism, host adhesion (Kern and Schneewind, 2008; Tarlovsky et al., 2010; Kern et al., 2012)]. Structure determination of the Sap SLH domain revealed that it comprises three SLH motifs that fold in a pseudo-trimer and that conserved positively charged residues sustain its interaction with the terminal PG-anchored pyruvylated-SCWP unit (Blackler et al., 2018; Sychantha et al., 2018). While the Sap and EA1 SLH domains are similar (74%), their AD are divergent (42% similar; 22% identity; Chateau et al., 2020).

In a recent study, we overcame hurdles concerning SLP self-polymerization and monomer stability. Using anti-Sap nanobodies (Nbs) as crystallization-aid (Muyldermans, 2013), we determined the first complete SLP AD structure of a pathogen, the Sap^AD^ (Fioravanti et al., 2019). Sap represents a novel class of SLPs that folds and assembles in a calcium-independent manner. Sap^AD^ folds into an extensive multi-domain protein consisting of six β-sandwich domains connected by short linkers. In solution, it adopts a flat tile-like supertertiary structure consisting of an “arm” (D1–2) and “body” (D3–6; Figure 1A). Interestingly, our recent comparison of the *B. anthracis* Sap^AD^ with the AD from SbsB from *Geobacillus stearothermophilus* revealed that the “arm-and-body” modular architecture is conserved across Bacillales (Fioravanti et al., 2019; Figure 1A). This architectural conservation is remarkable given the low average pairwise sequence identity (∼25%) and high variability in the domain ultrastructure. Moreover, the different proteins assemble into unrelated lattices and differ in their need for divalent metal ions for folding and S-layer assembly (Fioravanti et al., 2019). Further structural and functional insights are needed to understand better what governs the selective pressure(s) to maintain this “arm-and-body” architecture, despite the lack of structural conservation within and across domains and monomers within the S-layers.

### 2D Lattice

Electron microscopy has enabled an in-depth investigation of the lattice topology of native S-layer fragments and *in vitro* recrystallized S-layers (Couture-Tosi et al., 2002; Wang et al., 2015; Fioravanti et al., 2019). EM analysis on native S-layers resulted in low-resolution projection maps for both SLPs, with the density distribution hinting at six/seven domains for Sap and four for EA1 (Couture-Tosi et al., 2002). While recent EM studies on *in vitro* recrystallized S-layers show increased details about the Sap lattice (Fioravanti et al., 2019), the combined literature still does not reveal interdomain contacts responsible for Sap and EA1 S-layer assembly. In addition to the EA1 atomic structure, *in vitro* and on-cell cryo-EM/tomography studies leading to higher resolution density maps will be required to unveil details of the peculiar SLP switching mechanism, the *raison d’être* of the two S-layers, and avenues for therapeutic targeting of *B. anthracis* SLPs.

### Sap and EA1 as Vaccines or Antimicrobial Targets

At the outset, the contribution of the S-layer to *B. anthracis* virulence remains unclear. Deletion strains of either SLPs are viable *in vitro* but have never been tested under infection. Interestingly, the *sap* deletion mutant showed cell division defects due to a displacement of BslO, a SLAP that catalyses mother-daughter cell separation, which requires Sap S-layer for correct deposition at nascent cell division sites (Kern et al., 2012). In early 2000, Sap and EA1 were shown to be immunogenic during human infection (Baillie et al., 2003), nominating SLPs as potential vaccine candidates against anthrax. Subsequent studies have shown that immunization using EA1, but not Sap, offers a protective effect in a mouse model of inhalational anthrax (Uchida et al., 2012; Fioravanti et al., 2019).

Recently, Fioravanti et al. (2019) established a direct link between the *B. anthracis* S-layer integrity and its potential as an antimicrobial target. Anti-Sap-Nbs were shown to depolymerize the Sap S-layer *in vitro* and *in vivo*. *In vivo*, the Nbs-mediated disruption of the Sap S-layer resulted in severe morphological defects (wrinkled phenotype) and attenuated growth. The Nbs-induced phenotype was more striking than the *sap* knockout, suggesting that cells undergoing an acute loss of S-layer cannot adapt by switching to an EA1 S-layer to rescue such defects. These data point to a more critical contribution of S-layers in cell shape maintenance. Moreover, subcutaneous delivery of Sap-inhibitory Nbs cleared *B. anthracis* infection and prevented lethality in a mouse model of anthrax (Fioravanti et al., 2019). Together, these findings represent the first evidence that the disruption of S-layer integrity is a mechanism with therapeutic potential in S-layer-carrying pathogens.

Similarly, in *B. cereus* G9241, the causative agent of anthrax-like disease, mutants incapable of retaining Sap, EA1, and BSLs in the bacterial envelope showed reduced virulence in mice (Wang et al., 2013). Moreover, studies on S-layer distribution among the *B. cereus* group (containing *B. anthracis*) have observed SLPs in all clinical strains but only sporadically in environmental strains, suggesting a correlation between virulence and the presence of an S-layer (Mignot et al., 2001).

## The Two-Tiered Armor: *C. difficile* S-layer

*Clostridioides difficile* is an obligate anaerobic, spore-forming bacterium involved in a broad spectrum of diseases: from mild post-antibiotic diarrhea to severe pseudomembranous colitis, resulting in severe healthcare burden (Rupnik et al., 2009). CDC has designated *C. difficile* as the pre-eminent of five “Urgent Threats” to US healthcare, emphasizing its increasing antibiotic resistance [Centers for Disease Control and Prevention (CDC), 2019]. The *C. difficile* S-layer is shown to play a crucial role in the intestinal colonization step during infection (Calabi et al., 2002), in sporulation, toxin production, and resistance to components of the innate immune system (Kirk et al., 2017), representing an ideal candidate for the development of new therapeutics.

### S-layer Composition

The *C. difficile* S-layer represents a rare case where the 2D crystal is made by the assembly of heterodimers (Calabi et al., 2001). The *slpA* gene encodes for a common precursor (Karjalainen et al., 2001), which upon signal peptide removal and cell secretion, undergoes a second cleavage by the cysteine protease, Cwp84 (Kirby et al., 2009), releasing the high-molecular-weight (HMW) and the low-molecular-weight (LMW) SLPs. Together, they form a tightly-associated non-covalent H/L complex that anchors at the cell surface and assembles into the S-layer (Fagan et al., 2009; Figure 1B). A recent microscopy-based study revealed novel insights on subcellular SlpA secretion and S-layer growth. While S-layer growth occurs at specific sites that coincide with cell wall synthesis, the SLPs are secreted all over the cytoplasmic membrane, suggesting that there is a reservoir of SLPs within the cell wall ready to be utilized for S-layer growth (Oatley et al., 2020).

### Domain Organization and Structure

The SlpA precursor comprises an *N*-terminal signal peptide, the LMW-SLP, and the *C*-terminal HMW-SLP (Calabi et al., 2001; Figure 1B). The HMW-SLP is anchored to SCWP anionic polymer PSII by the cell wall binding domain 2 (CWB2; Figure 1B), while the LMW-SLP is presented as the outermost component of the *C. difficile* surface, showing a high degree of antigenic variation between strains (Calabi et al., 2001; Willing et al., 2015). CWB2 comprises three tandem motifs (Willing et al., 2015) as seen for SLH (Kern et al., 2011; Figure 1B). Despite being similar in sequence, the CWB2 motifs are not redundant; it takes three motifs to ensure the S-layer anchoring to the cell wall (Willing et al., 2015). *C. difficile* encodes an additional 28 CWB2 carrying SLAPs, called the clostridia cell wall proteins (CWPs; Fagan et al., 2011). As with Bacillus *BSLs*, CWPs do not form the S-layer but exploit a variety of enzymatic and host-pathogen interaction functions (Kirby et al., 2009; Bradshaw et al., 2017). Recent structure determination of Cwp8 unveiled the CWB2 domain fold (Usenik et al., 2017; Figure 1B). Each CBW2 motif assumes a topoisomerase-primase fold, and together they assemble in a trefoil-like shape (Figure 1B). EM studies revealed the presence of a two-tiered S-layer at the cell surface (Cerquetti et al., 2000). Determining the atomic structure of the SlpA heterodimer or in its S-layer form has proven challenging. The crystal structure of an LMW-SLP truncated version was determined (Figure 1B); the missing 59 *C*-terminal residues were reported as necessary for heterodimer formation (Fagan et al., 2009). The LMW-SLP assumes a novel fold comprising two domains: D1 contains both the *N*- and *C*-termini of the protein that fold into a sandwiched conformation; D2, likely exposed at the cell surface, presents a novel fold with a high loop content. The loops allow a high-level of sequence variability that promotes host immune system evasion while retaining the overall SLP fold (Fagan et al., 2009; Spigaglia et al., 2011; Merrigan et al., 2013). Small-angle X-ray scattering was used to study the H/L complex. In solution, the two SLPs are arranged in an “end-to-end” complex with presumably the *C*-terminus of LMW-SLP and *N*-terminus of HMW-SLP interacting with each other. A recent preprint describes the SlpA S-layer organization in atomic detail (PDB: 7ACY; Banerji et al., 2021). In this structure, the LMW/HMW SLP-interacting domains are described to fold into a “paper-clip” arrangement, while the three CWB2 motifs of the HMW subunit are organized in a triangular prism. Moreover, the crystallographic structure of the H/L heterodimer could be docked in the EM projection maps obtained on native SlpA S-layer, unveiling important intramolecular interfaces essential for S-layer formation. This work represents a significant advancement for the S-layer and *C. difficile* communities, offering a plethora of possibilities for the design of S-layer-structure-tailored antimicrobials (Banerji et al., 2021).

### SlpA and Virulence

SlpA is required for gastrointestinal tissue adherence and is implicated in pathogenicity (Calabi et al., 2002; Merrigan et al., 2013). SLP mutants have been impossible to obtain, suggesting the essentiality of the *slpA* gene. Instead, two rare resistant mutants to diffocin, a bacteriocin that selectively kills *C. difficile* strains, displayed an SLP-null phenotype that presents severe sporulation defects and a significant increase in bacterial susceptibility to lysozyme and the antimicrobial peptide, LL-37 (Kirk et al., 2017). Interestingly, these mutants are capable of colonizing the intestinal tract of hamsters despite a complete attenuation of virulence. SLPs are also found in several other Clostridia species, including *Clostridium botulinum* (Takumi et al., 1992) and *Clostridium tetani* (Sleytr and Messner, 1983; Takumi et al., 1991). Further characterization and comparative studies are needed to delineate the SLP biology in other Clostridia pathogens.

## Other Gram-Positive “Flavors” of Slps

In addition to the well-characterized Bacilli and Clostridia SLPs, several pathogenic members within Firmicutes (e.g., Paenibacilli, Lactobacilli, and Listeria), as well as Actinobacterial species (e.g., Corynebacteria, Mycobacteria), are known to form S-layers linked to their virulence and pathogenicity (Sleytr and Messner, 1983; Fagan and Fairweather, 2014). In this section, we highlight a few Gram-positive variations, with and without SLH/CWB2 domains (Figure 2).

### Paenibacilli

Many Paenibacilli, including the etiological agent of the epizootic of honeybees *P. larvae*, possess a functionally proven virulent S-layer made of SlpA (Poppinga et al., 2012). *P. alvei* cells present an S-layer comprised of glycosylated SLP, SpaA. In these SLP homologs, the *N*-terminal SLH domain has dual recognition for SCWP and PG, and is sufficient for *in vivo* cell surface display of foreign proteins at the cell surface (Janesch et al., 2013; Figure 2). The SLH domain trimer rearrangement also relieves any S-layer strain caused by cell growth and division (Blackler et al., 2018). Notably, a second SLH-containing protein in *P. alvei*, SlhA, is found to be vital for swarming and biofilm formation (Janesch et al., 2013).

### Other Pathogenic Firmicutes

Other notable Firmicutes such as Streptococcus, Staphylococcus, and Listeria have been predicted to carry SLPs (Figure 2) that have been indirectly linked to pathogenicity (Navarre and Schneewind, 1999; Camejo et al., 2009; He et al., 2019). For instance, studies involving *L. monocytogenes* virulence factors suggest a role for S-layer glycoproteins in Listeria virulence (Camejo et al., 2009). Further structural and functional characterizations remain to be performed.

### Non-Pathogenic Lactobacillales

One of the rarer symbiotic functional contexts in which S-layers have been reported is in the Lactobacilli and Enterococcus species that adhere to intestinal epithelial cells. Several of these species contain non-glycosylated SLPs with an SlpA domain for cell anchoring instead of the typical SLH domain. While few Lactobacilli species carry multiple copies of SLPs (Åvall-Jääskeläinen et al., 2008; He et al., 2019), probiotic strains carry SLAPs that contain collagen- and fibronectin-binding domains, which are useful to adhere to the extracellular matrix of the intestinal epithelial cells (Bahl et al., 1997; Hymes et al., 2016) and contribute to pathogen exclusion (Martínez et al., 2012). In other species such as *L. crispatus*, SlpB interacts with the bacterial cell wall, and its collagen-binding activity is thought to aid in antigenic variation in adherence (Bahl et al., 1997) and gut colonization (Sun et al., 2017).

### Corynebacteria, Mycobacteria

Even actinobacterial species with atypical outer membrane-like structures (known as Mycomembrane) carry SLPs. For instance, few strains of *C. glutamicum* contain a hexagonal S-layer made of PS2 (Bahl et al., 1997; Chami et al., 1997; Houssin et al., 2002; Burkovski, 2013). While PS2’s *N*-terminus is responsible for monomer interactions, its *C*-terminal region, especially with a hydrophobic stretch, is needed for cell wall anchoring (Bahl et al., 1997; Bayan et al., 2003). In contrast, S-layers with an oblique arrangement have been reported in Mycobacterial species such as *M. bovis* (Lounatmaa and Brander, 1989). The highly immunogenic nature of a few mycobacterial CWPs suggests that the cell wall antigens are located in the S-layer (Lounatmaa and Brander, 1989).

## Conclusion

S-layers are paracrystalline protein arrays that are among the most commonly observed cell envelope components in prokaryotes. They are important for cell development, cell-environment, and cell-host interactions (Fagan and Fairweather, 2014). Bacterial SLPs exhibit considerable variation in their composition and structure, as evident from the low sequence similarities across SLP homologs (Sleytr and Messner, 1983; Bahl et al., 1997; Navarre and Schneewind, 1999; Sleytr and Beveridge, 1999; Fagan et al., 2009; Kufel et al., 2017). Typically, Gram-positive SLPs comprise an *N*-terminal signal peptide, a cell wall anchoring domain, and an AD that self-polymerizes into the S-layer (Figures 1, 2). In this mini-review, we discuss recent breakthroughs in S-layer structure and function in two prominent Gram-positive pathogen-containing classes, Bacilli and Clostridia (Figure 1; Kirk et al., 2017; Fioravanti et al., 2019; Oatley et al., 2020; Banerji et al., 2021) accentuating the critical role played by S-layer in cell envelope integrity and bacterial pathogenicity. We also highlight notable variations of S-layers in other Firmicutes and Actinobacteria, with the responsible proteins containing lineage-specific SLP (SLH, CWB2, SlpA, and LMW-SLP) and paralogous SLAP (e.g., amidase, fibronectin, adhesin, and endotoxin) domains (Figure 2). With no apparent sequence signature, finer evolutionary analyses on SLPs and SLAPs across bacterial and archaeal phyla are required to shed light on their evolutionary origin and function.

In summary, S-layers represent the frontline for host-pathogen interactions playing a significant role in virulence and modulating the host immune response. Because they are abundant pathogen-specific components, exposed on the cell surface, SLPs can be exploited as diagnostic, vaccine, and therapeutic targets. The advent of new experimental and computational technologies will open new avenues to further characterize the currently unresolved sequence-structure-function links in these extraordinary macromolecular scaffolds.

## Author Contributions

JR and AF contributed equally to the conceptualization and the writing of this manuscript. Both authors contributed to the article and approved the submitted version.

## Conflict of Interest

The authors declare that the research was conducted in the absence of any commercial or financial relationships that could be construed as a potential conflict of interest.
